# Determinants of participation restriction among community dwelling stroke survivors: A path analysis

**DOI:** 10.1186/1471-2377-9-49

**Published:** 2009-09-07

**Authors:** Janita PC Chau, David R Thompson, Sheila Twinn, Anne M Chang, Jean Woo

**Affiliations:** 1 Nethersole School of Nursing, Chinese University of Hong Kong, Hong Kong; 2 Department of Health Sciences, University of Leicester, Leicester, UK; 3 Queensland University of Technology and Mater Health Services, Australia; 4 School of Public Health, Chinese University of Hong Kong, Hong Kong

## Abstract

**Background:**

Apart from promoting physical recovery and assisting in activities of daily living, a major challenge in stroke rehabilitation is to minimize psychosocial morbidity and to promote the reintegration of stroke survivors into their family and community. The identification of key factors influencing long-term outcome are essential in developing more effective rehabilitation measures for reducing stroke-related morbidity. The aim of this study was to test a theoretical model of predictors of participation restriction which included the direct and indirect effects between psychosocial outcomes, physical outcome, and socio-demographic variables at 12 months after stroke.

**Methods:**

Data were collected from 188 stroke survivors at 12 months following their discharge from one of the two rehabilitation hospitals in Hong Kong. The settings included patients' homes and residential care facilities. Path analysis was used to test a hypothesized model of participation restriction at 12 months.

**Results:**

The path coefficients show functional ability having the largest direct effect on participation restriction (β = 0.51). The results also show that more depressive symptoms (β = -0.27), low state self-esteem (β = 0.20), female gender (β = 0.13), older age (β = -0.11) and living in a residential care facility (β = -0.12) have a direct effect on participation restriction. The explanatory variables accounted for 71% of the variance in explaining participation restriction at 12 months.

**Conclusion:**

Identification of stroke survivors at risk of high levels of participation restriction, depressive symptoms and low self-esteem will assist health professionals to devise appropriate rehabilitation interventions that target improving both physical and psychosocial functioning.

## Background

Stroke is the second leading cause of death globally, and the human and economic consequences are profound. According to the Global Burden of Disease Report, stroke is the third leading cause of disease burden for high-income countries, and the seventh for low to middle-income countries. [1] Lack of social contact or social isolation are common sequelae of stroke due to cognitive and physical impairments and communication disorders. [2] One-fifth of patients who survive stroke require institutional care for the remainder of their lives [3] and around one-third require rehabilitation services and long term care support. [4] Thus, whether returning home or moving into residential care after the acute event, ongoing community support for stroke survivors is essential.

Apart from promoting physical recovery and assisting in activities of daily living, a major challenge in stroke rehabilitation is to minimize psychosocial morbidity and to promote the reintegration of stroke survivors into their family and community. The World Health Organization (WHO) framework of Functioning, Disability and Health highlights the importance of people with a health condition functioning in society. [5] This often necessitates social integration, return to work potential and work performance. The measurement of participation gives a more objective view of recovery that is important in estimating recovery. [6] Psychosocial factors of concern in the longer-term outcome of participation after stroke include depression, self-esteem, and social support. An emphasis on these as well as recovery of functional ability provides a more complete picture of the experiences of patients following stroke. [5,7] Thus the aim of this study was to test a theoretical model of predictors of participation restriction which included the direct and indirect effects between psychosocial outcomes, physical outcome, and socio-demographic variables at twelve months after stroke. The identification of key factors influencing long-term outcome are essential in developing more effective rehabilitation measures for reducing stroke-related morbidity.

## Methods

### Design, Setting and Sample

The findings presented here are part of a longitudinal study. Data were collected from 188 stroke survivors at 12 months following their discharge from one of the two rehabilitation hospitals in Hong Kong (attrition rate: 29% over 12 months). Originally, these acute stroke patients had transferred for rehabilitation from acute hospitals in one geographical region. The multi-disciplinary rehabilitation programme comprised medical and nursing care, physiotherapy and occupational therapy in the rehabilitation hospitals and patients were, if necessary, seen by a medical social worker and/or psychologist. The average length of stay in the rehabilitation hospitals ranged from two to three weeks. Data collection took place at 12 months in either the patient's home or other discharge destination such as a residential care facility.

### Inclusion and Exclusion Criteria

Patients with a diagnosis of stroke were included in the study. Stroke was diagnosed by a neurologist and stroke types were classified according to the results of neuro-imaging i.e., supported or confirmed by computerized tomography (CT) or magnetic resonance imaging (MRI). The inclusion criteria were that patients had a score of 18 out of a possible 30 for the Mini Mental State Exam (MMSE), were a resident of Hong Kong, and could communicate in and be able to understand Cantonese. The study included patients with a first-ever stroke and those with a history of previous stroke in order to examine the influence of number of stroke episodes on post-stroke outcome.

### Hypothesis

We hypothesized that state self-esteem, depressive symptoms, functional ability, social support satisfaction and stroke survivors' socio-demographic variables influence participation restriction at 12 months.

### Measures

Participation restriction: the London Handicap Scale (LHS), [8] used to measure restriction in participation, is a 6-item tool and for each of the 6 dimensions of handicap respondents rate the extent (0 = extreme disability and 5 = no disability) to which their level of health inhibits them from performing the activity: getting around, looking after yourself, work and leisure, getting on with people, awareness of your surroundings, affording the things you need. High scores on the LHS indicate low participation restriction. The LHS was translated into Chinese and in a Hong Kong study, [9] there was a significantly positive correlation between the mean ratings of the translated version of LHS scores between Hong Kong and the UK subjects (r = 0.87, p = 0.001). In this study the Cronbach alpha was 0.80.

State Self-esteem: the State Self-esteem Scale (SSES) comprises 20 items. [10] Patients rate whether each item is true of themselves "right now", using a 5-point Likert scale with 1 = not at all, and 5 = extremely, yielding a total possible score of 20-100, with high scores indicating higher levels of state self-esteem. The SSES has a high internal consistency with an α of 0.92 in Heatherton & Polivy's study and in a Hong Kong study of stroke patients, the α for the Chinese SSES was 0.85. [11] In this study the Cronbach alpha for the SSES was 0.89.

Depressive symptoms: the Geriatric Depression Scale (GDS) is a 30-item scale with yes/no answers, a score of 11 indicating mild depression and a score of 17 severe depression. [12] The scale is used extensively as a clinical screening tool and research instrument in Western and Chinese stroke populations and has good psychometric properties. [13] The Chinese (Cantonese) back-translated version of the GDS was validated and the alpha coefficient for the Chinese GDS was high at 0.89 and the test-retest reliability coefficient was 0.85. [14] In this study the Cronbach alpha for the GDS was 0.92.

Social support: the Social Support Questionnaire (SSQ6) was used to determine the quantity of support each patient had as well as their satisfaction with the support provided. [15] Respondents indicate from 0-9 the number of support persons they have for the six situations (number score) and rate their overall satisfaction with the support provided, using a 6-point Likert scale (satisfaction score), higher SSQ6 satisfaction scores being indicative of more satisfaction with social support received. The alpha coefficient for the Chinese versions of the SSQ6-number was 0.87 and the SSQ6-satisfaction - 0.92. [11] In this study the Cronbach alpha for the SSQ6-satisfaction was 0.95.

Functional ability: the Modified Barthel Index (BI) [16] is designed to assess the degree of independence a patient has in performing the various self-care and mobility activities of daily living (ADL) tasks. [17] It comprises 15 items rated on 3-point scale: "Can do by myself", "Can do with help of someone else" and "Cannot do at all" with predetermined scores according to which of the three ratings is selected, with 0 for the "Cannot do at all" rating. The total possible score ranges from 0 (total dependence) to 100 (total independence). In this study the Cronbach alpha for the BI was 0.90.

Socio-demographic and disease specific information: Age, gender, marital state, educational level, occupation, religion, comorbidity, type and number of strokes and living arrangement were collected.

### Procedures

Approval had been obtained from the university and hospital ethics committees. All patients meeting the inclusion criteria were invited to participate following an explanation of the purpose and were informed about their rights and freedom to withdraw from the study at any time without it influencing their treatment. If they agreed to participate they were asked to sign a consent form. All patients agreeing to participate were interviewed at 12 months following discharge from the rehabilitation hospitals.

### Statistical Analysis

Path analysis is a statistical technique that uses both bivariate and multiple linear regression techniques to test the causal relations among the variables specialized in the model. [18] Path coefficients were computed via a series of multiple regression analyses based on the hypothesized model. The colinearity of the data was checked using the colinearity diagnostics in SPSS. Path diagrams were constructed with a single headed arrow representing the causal order between two variables, with the head pointing to the effect and the tail to the cause. A curved, double arrow indicating a correlation between two variables. [18] The sample size in this study was adequate based on the recommendation by Kline [19] that 10-20 times as many cases as parameters is sufficient for significance testing of model effects.

## Results

Most of the participants were male (61.7%), married (70.2%), and had received primary or less than primary school education (74.5%). Eighty-one (43.1%) had a right hemisphere lesion. Ages ranged from 45 to 91 (mean, 71.7; SD, 10.2) years. Thirty-two (17%) of the survivors were in a residential care facility. The mean BI score at 12 months was 85.9. For 31 (16.5%) patients at 12 months the BI was 60 or below, indicating that they were markedly dependent in self care and mobility. [20]

The hypothesis predicting that state self-esteem, depressive symptoms, functional ability, social support satisfaction and socio-demographic variables influence participation restriction at 12 months was tested with path analysis. Participation restriction was the dependent variable. Exogenous independent variables were age, functional ability, living arrangement and gender. Endogenous independent variables were depressive symptoms, state self-esteem and social support satisfaction. No problem of multi-colinearity was detected as bivariate correlations did not exceed 0.80. [21] Residual plots were used to check normality and no violation of assumption of normality was detected. Path coefficients were calculated via a series of multiple regression analyses based on the hypothesized model and the results are presented in Table 1.

**Table 1:** Path Coefficients Calculated via a Series of Multiple Regression Analyses based on the Hypothesized Model

**Outcome variables**	**R**^2^	**Predictor variables**	**β**	***p***
Participation restriction	0.71	**Depressive symptoms**	-0.266	<0.001
		**State self-esteem**	0.185	0.013
		Social support satisfaction	0.019	0.739
		**Functional ability**	0.512	<0.001
		**Age**	-0.111	0.009
		**Living arrangement**	-0.116	0.008
		**Gender**	0.127	0.003
Depressive symptoms	0.60	**State self-esteem**	-0.723	<0.001
		**Functional ability**	-0.108	0.038
		**Age**	-0.104	0.031
		Living arrangement	0.083	0.099
State self-esteem	0.59	**Depressive symptoms**	-0.759	<0.001
		**Age**	-0.116	0.017
		Living arrangement	0.023	0.642

Note: Predictor variables with a significant level < 0.05 are bold and retained in the final model.

High scores on the LHS indicate low participation restriction.

As predicted, depressive symptoms, state self-esteem, functional ability, living arrangement, age and gender had a direct significant effect on participation restriction. However, no significant direct relationship between social support satisfaction and participation restriction was found.

For the final model, non-significant variables from regression models were deleted and repeated with only the significant variables (Table 2). Standardized regression beta weights were used to calculate the direct (the influence of one variable on another that is not medicated by any other variable in a model) and indirect (the effect of one variable on another through at least one other variable in a model) effects of significant variables on participation restriction. [18]

**Table 2:** Final Model of Participation Restriction (n = 188)

**Outcome variables**	**R**^2^	**Predictor variables**	**β**	***p***
Participation restriction	0.71	Depressive symptoms	-0.268	<0.001
		State self-esteem	0.198	0.002
		Functional ability	0.512	<0.001
		Age	-0.109	0.010
		Living arrangement	-0.116	0.008
		Gender	0.129	0.002
Depressive symptoms	0.59	State self-esteem	-0.728	<0.001
		Functional ability	-0.134	0.008
		Age	-0.096	0.047
State self-esteem	0.59	Depressive symptoms	-0.754	<0.001
		Age	-0.113	0.018

Note: High scores on the LHS indicate low participation restriction.

**Figure 1:**
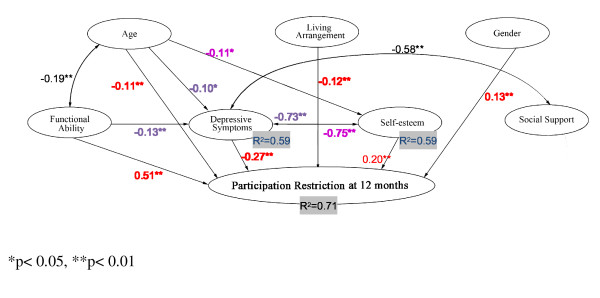
**Trimmed model with path coefficients at 12 months**.

According to the trimmed model with path coefficients at 12 months (see Figure 1), the path coefficients show functional ability having the largest direct effect on participation restriction (β = 0.51). The results also show that more depressive symptoms (β = -0.27), low state self-esteem (β = 0.20), female gender (β = 0.13), older age (β = -0.11) and living in a residential care facility (β = -0.12) have a direct effect on participation restriction. The explanatory variables accounted for 71% of the variance in explaining participation restriction at 12 months.

Living arrangement had no indirect effect on participation restriction through depressive symptoms and state self-esteem. Age (β = -0.10), functional ability (β = -0.13), state self-esteem (β = -0.73) had significant negative indirect effect on participation restriction through their effects on depressive symptoms. Depressive symptoms appear to be most strongly affected by state self-esteem and the association with state self-esteem was to a much greater degree than functional ability. The model variables account for 59% of the variance of depressive symptoms. Age (β = -0.11) and depressive symptoms (β = -0.75) also had negative indirect effects on participation restriction through their effects on state self-esteem and the model variables account for 59% of the variance of state self-esteem.

An alternative model of participation restriction was constructed and repeated with depressive symptoms, functional ability, living arrangement, age and gender due to the high interdependence of state self-esteem and depressive symptoms. This alternative model resulted in an R^2 ^of 0.69 which was close to 0.71 for the original model used in this study. The results showed that functional ability, depressive symptoms, age, living arrangement and gender continued to predict participation restriction when state self-esteem was excluded. According to the alternative model (Table 3), functional ability had the largest direct effect on participation restriction (β = 0.52). The results showed that more depressive symptoms (β = -0.41), female gender (β = 0.11), older age (β = -0.13) and living in a residential care facility (β = -0.11) had a direct effect on participation restriction. The explanatory variables accounted for 69% of the variance in explaining participation restriction at 12 months.

**Table 3:** Alternative Model of Participation Restriction (without a measure of State Self-esteem) (n = 188)

**Outcome variables**	**R**^2^	**Predictor variables**	**β**	***p***
Participation restriction	0.69	Depressive symptoms	-0.413	<0.001
		Functional ability	0.519	<0.001
		Age	-0.130	0.002
		Living arrangement	-0.111	0.014
		Gender	0.106	0.012

Note: High scores on the LHS indicate low participation restriction.

## Discussion

### Participation Model

Participation restriction is concerned with the social aspects resulting from disability in terms of an individual's level of participation in life roles. The International Classification of Functioning, Disability and Health challenges mainstream ideas on how people understand health and disability (biomedical model), and takes into account the social aspects of disability. [5] Although this framework provides a broader conceptual framework for understanding health and disability, further conceptual development is required to facilitate understanding of the participation restriction under various health conditions. Prediction models have empirical and practical applications [22] such as suggesting important factors to be considered and helping develop targets in promoting stroke recovery. Models exist in predicting functional recovery after stroke, [23] however, to date, no path analysis of the prediction of participation restriction in stroke population has been published. Path analysis was used in this study to test a hypothesized model of participation restriction at 12 months to guide practice and provide direction for future research. Path analysis is superior to ordinary regression analysis as it provides an explanation of both the casual relation and the relative importance of alterative paths of influence. [18] This model tries to capture the complex dynamics of participation restriction in life roles by incorporating biological and psychosocial aspects.

#### Direct effects

The path coefficients show that functional ability has the largest direct effect and explains the most variance on participation restriction. An increase of one standard deviation in functional ability produces an increase of 0.51 standard deviation in participation level. [18] The result also supports the notion that more depressive symptoms and low state self-esteem have a direct effect on participation restriction. Socio-demographic (older age, female gender) and contextual (living arrangement) factors also have a direct effect on participation restriction. The explanatory variables accounted for substantial proportions of variance (71%) in explaining participation restriction at 12 months. Functional ability has the largest direct effect on participation restriction and this is consistent with another study. [24]

Similar to previous work, the factors predicting the level of participation restriction in long- term post-stroke survivors were physical function, depression, [24-26] age, [26,27] and living arrangement. [25]

Advanced age is consistently identified in the literature [26] and in this study as being related to physical and psychosocial morbidity. Older individuals in this study also had poor functional ability which is likely to hinder their resumption of social roles in day-to-day life. Consistent with other studies, [28,29] female gender was associated with higher levels of participation restriction. Female participants in this study also had a significantly lower self-esteem scores when compared with male participants, which is consistent with previous research. [30,31] Female stroke survivors were also found to be less likely to engage in post-stroke social and leisure activities and it might be explained by the great value placed on body image by women. [32] In this study, those who lived in residential care facilities had a significantly higher level of participation restriction, lower state self-esteem, and a higher level of depressive symptoms. This highlights major challenges for health professionals who care for stroke survivors in residential care facilities. Being institutionalized was found to be associated with participation restriction among a group of 95 stroke survivors follow-up 12 months after stroke. [29] Further studies that investigate the associations between environmental barriers, psychological morbidity and the occurrence of participation restriction following a stroke among stroke survivors are needed.

The path analysis model shows that all variables, apart from social support satisfaction, have a direct effect on participation or were mediated through depressive symptoms. The lack of a direct association between social support satisfaction and participation restriction is a finding that is consistent with other studies. [11] The result could be due to social support being generally high in the current study with little variance. The model, accounting for 71% of the variance in participation restriction, suggests that other variables should be included to more fully explain the outcome. Future model construction and testing could incorporate other measures to assess, for example, the degree of communication impairment, [33] appraisals and coping, [34] and environmental barriers [35] that could affect participation.

#### Indirect effects

The final path model shows age, functional ability, and state self-esteem had a significant negative indirect effect on participation restriction through their effects on depressive symptoms. Depressive symptoms appear to be most strongly affected by state self-esteem and the association with state self-esteem was to a much greater degree than functional ability. Age and depressive symptoms also had negative indirect effects on participation restriction through their effects on state self-esteem.

## Conclusion

This study, using a broader, more inclusive framework to assess post-stroke outcomes, indicates that stroke survivors in the first year after discharge face difficulties in participating in activities essential for social and community life. The study identifies important factors to be considered in helping adaptation and in promoting recovery. These factors accounted for 71% of the variance in explaining participation restriction. The above findings indicates that rehabilitation services need to continue to focus on restoring functional independence but also need to diagnose and treat depressive symptoms in order to minimise the restriction to participation in society. Assisting stroke survivors in redefining their identity after stroke could be an important aspect in stroke rehabilitation. Improving communication with stroke survivors and carers, and avoiding categorising stroke survivors by their deficits, [36,37] could help to enhance stroke survivors' self-esteem and in turn contribute to the societal participation.

There are a number of limitations to this study. The sample excluded those who were cognitively impaired, with MMSE scores less than 18. The linguistic demand of the instruments also excluded those stroke survivors with communication difficulties that bear the greatest burden of the morbidity. Consequently, the results of this study cannot be extrapolated to all stroke survivors seen in general clinical practice. The method of recruiting patients in this study was that of convenience sampling, and data were obtained from patients from two rehabilitation hospitals; generalization of these findings might thus be limited. The attrition rate of 29% over 12 months also reduced the ability to generalize the findings. With regard to educational level, only about one quarter of the participants in this study had secondary or higher education due to the lack of available secondary education for this group in the 1950s when these patients were still young and the findings need to be interpreted in this context.

## Competing interests

The authors declare that they have no competing interests.

## Authors' contributions

JPCC conceived of the study, and participated in its design and coordination, performed the statistical analysis and drafted the manuscript. DRT helped to draft the manuscript. ST reviewed the manuscript. JW participated in its design. AMC conceived of the study and participated in the design of the study. All authors read and approved the final manuscript.

## Pre-publication history

The pre-publication history for this paper can be accessed here:


